# Enhancing Quasi‐Two‐Dimensional Electronic Structure in Bi_2_O_2_Se via Chlorine Substitution

**DOI:** 10.1002/advs.77919

**Published:** 2026-09-27

**Authors:** Yong‐Zhen Jiang, Qi‐Xun Wen, Xiang Xu, Su‐Tao Sun, Feng‐Fan Ren, Zi‐Xuan Zhou, Xing‐Yuan You, Yu‐Cheng Kan, Zheng Zhang, Jie‐Yi Liu, Yu‐Zhou Zhao, Zheng‐Tai Liu, Mao Ye, Shu‐Hua Yao, Jian Zhou, Yan‐Bin Chen, Zhong‐Kai Liu, Yu‐Lin Chen, Yang‐Yang Lv, Cheng Chen

**Affiliations:** ^1^ State Key Laboratory of Quantum Functional Materials and ShanghaiTech Laboratory For Topological Physics School of Physical Science and Technology ShanghaiTech University Shanghai China; ^2^ National Laboratory of Solid State Microstructures Department of Materials Science and Engineering Department of Physics Nanjing University Nanjing China; ^3^ Diamond Light Source Harwell Science and Innovation Campus Didcot UK; ^4^ Shanghai Synchrotron Radiation Facility Shanghai Advanced Research Institute Chinese Academy of Sciences Shanghai China; ^5^ National Key Laboratory of Materials for Integrated Circuits Shanghai Institute of Microsystem and Information Technology Chinese Academy of Sciences Shanghai China

## Abstract

Bismuth oxyselenide (Bi_2_O_2_Se) has emerged as a leading semiconductor candidate for next‐generation electronics and quantum phenomena research. However, its strong ionic interlayer bonding of Se layers fundamentally restricts the full potential of this otherwise promising quasi‐two‐dimensional (2D) material. In this work, we demonstrate chlorine substitution as an effective strategy to introduce van der Waals gaps and modify the electronic structure of the system. Through comprehensive transport and angle‐resolved photoemission spectroscopy (ARPES) measurements on Bi_6_O_6_Se_2_Cl_2_ single crystal, an optimal Cl‐doping compound, we show that the key electronic features, including indirect bandgap, high electron mobility, and carrier effective mass, are well preserved, while the 2D character of the system is sufficiently enhanced. First‐principles calculations attribute this selective tunability to the unique spatial separation between conductive Bi‐O layers and dopant Se/Cl layers, which enables independent optimization of interlayer coupling and intralayer transport. Remarkably, our theoretical calculations combined with experimental observations project a broad bandgap engineering across a wide range (0.8 ∼ 3.5 eV) within the Bi_2_O_2_Se system. Our findings not only expand the application potential of Bi_2_O_2_Se‐based materials in optoelectronics, nanoelectronics, and fundamental research but also highlight spatial decoupling as a viable strategy for modifying the detailed electronic structure of quasi‐2D quantum systems.

## Graphical abstract

We demonstrate chlorine substitution as an effective strategy to transform Bi_2_O_2_Se into an exfoliatable van der Waals superlattice. Key electronic band feature, including indirect bandgap, effective mass is well preserved, while the 2D character of the system is sufficiently enhanced. This work highlights the spatial decoupling of the layers as an effective strategy for modifying the detailed electronic structure of quasi‐2D materials.

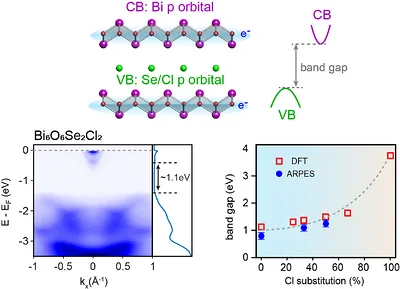

## 1 Introduction

Among the emerging 2D materials that possess exceptional properties, the quasi‐2‐dimensional (2D) bismuth oxyselenide (Bi_2_O_2_Se) has rapidly distinguished itself for its considerable potential in both industrial applications and fundamental physics research [1]. It features exceptional electronic properties with great environmental stability, including a moderate bandgap (∼0.8 eV), ultrahigh intrinsic electron mobility (20,000 cm^2^V^−^^1^s^−^^1^ at cryogenic temperatures), robustness of electronic properties against surface perturbations [2, 3]. These properties, together with their inherent ability to form native high‐permittivity (high‐κ) oxide‐insulator (Bi_2_O_2_Se/Bi_2_O_5_Se, mimicking Si/SiO_2_) [4, 5, 6], make it a leading candidate as the post‐silicon materials to sustain Moore's law. The construction of high‐performance Fin‐FET and gate‐all‐around FET (GAAFET) showcase its immediate potential for next‐generation electronic devices [7, 8]. Low‐cost and broad‐range photodetector with high responsivity and detectivity has also been constructed based on this material, illustrating its wide industrial application potential [9, 10, 11]. More importantly, the superior electronic properties of Bi_2_O_2_Se also make it a versatile platform for fundamental physics research. The compensation of Rashba spin polarization in adjacent Bi‐O layers (hidden Rashba effect) leads to the realization of unique quantum hall states (QHS), where only the even‐integer states present [12, 13, 14]. Notably, this even‐odd QHS transition could be further manipulated through field effect tuning [13], showcases its great potential in the investigation of fundamental quantum phenomena as well as future spintronics devices.

**FIGURE 1:**
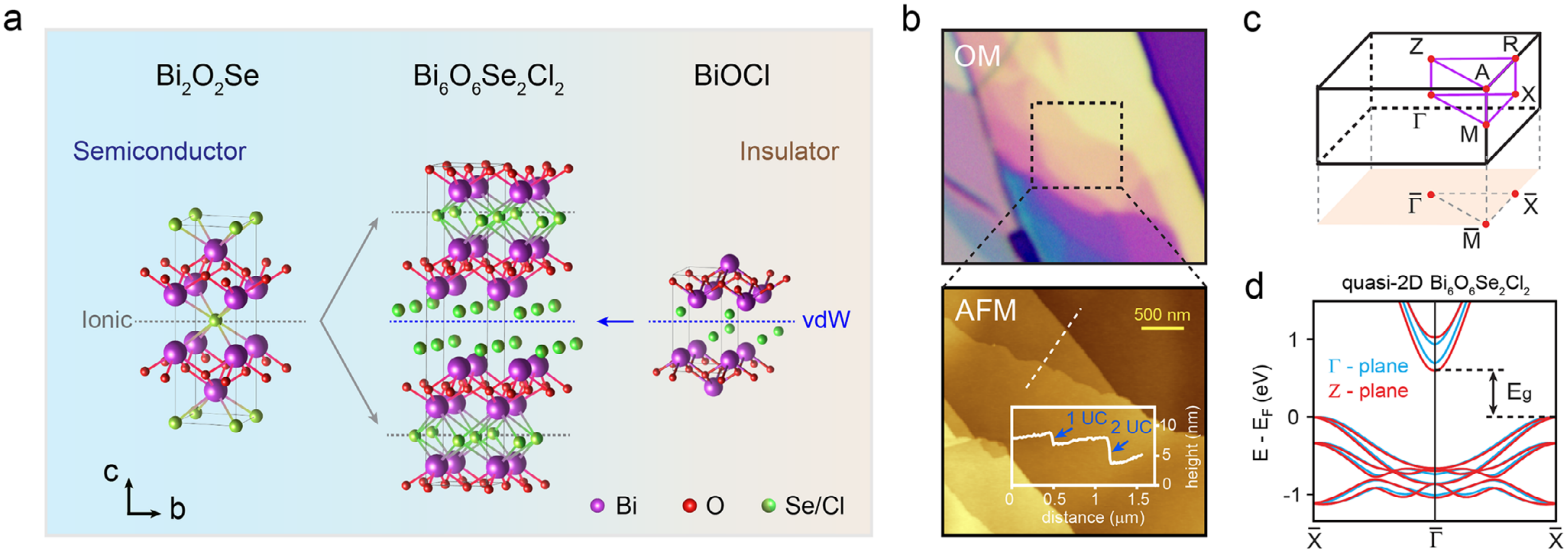
Crystal structure and quasi‐2D properties of Bi_6_O_6_Se_2_Cl_2_. (a) Comparison of the crystal structures of Bi_2_O_2_Se, Bi_6_O_6_Se_2_Cl_2_, and BiOCl. The substitution of Se by Cl atoms introduces van der Waals (vdW) gaps into the lattice. (b) Optical microscope (OM) and atomic force microscope (AFM) images of mechanical exfoliated thin flake of Bi_6_O_6_Se_2_Cl_2_. Height profiles (inset) confirm step heights corresponding to 1‐ and 2‐unit cells. (c) Brillouin zone and its projection onto the (001) surface with high‐symmetry points labeled. (d) Calculated band dispersions along the Γ and *Z* planes. The nearly identical dispersions indicate the quasi‐2D electronic nature of the system.

Despite its remarkable potential, an inherent limitation of the Bi_2_O_2_Se crystal structure restricts its broader application. Unlike conventional van der Waals materials, where weakly interacting layers enable facile mechanical exfoliation, Bi_2_O_2_Se is a quasi‐2D material—its conductive Bi_2_O_2_^2^^+^ layers are strongly bonded through bridging Se^2^^−^ anions, forming robust ionic interactions along the out‐of‐plane direction (Figure 1a). Consequently, obtaining high‐quality ultrathin Bi_2_O_2_Se requires specialized growth techniques such as molecular beam epitaxy (MBE) [12, 15], limiting its accessibility for device fabrication. The introduction of van der Waals gaps into this system could sufficiently expand its research opportunities both in application and fundamental research. For instance, mechanical exfoliation and stacking would enable the heterostructure engineering study through proximity‐induced phenomena and moiré potential engineering [16]. Reducing the interlayer coupling would improve electron performance while allowing better electrostatic control—crucial for making smaller transistors and quantum devices. In addition, enhancing 2D character would potentially accentuate quantum effects through confinement, where cleaner signals of quantum hall effects and stronger electron‐electron correlations are expected. Previous research efforts brought up a feasible approach to address this issue through chlorine substitution of selenium; however, limited in the form of powder or polycrystals [17, 18, 19, 20, 21]. A complete substitution is also not favored since it transforms the system into a wide‐bandgap insulator, BiOCl (∼ 3.5 eV) [17], sacrificing the exceptional electronic properties that make Bi_2_O_2_Se technologically valuable.

In this work, we report the synthesis and systematic electronic characterization of high‐quality Bi_6_O_6_Se_2_Cl_2_ single crystals, an optimal chlorine doping that both enables mechanical exfoliation through the introduction of a van der Waals gap and preserves the basic electronic properties of the system. Combining the use of angle‐resolved photoemission spectroscopy (ARPES) and transport measurement, we confirm that the key electronic features of Bi_2_O_2_Se are maintained, while the two‐dimensionality of the band structure is significantly enhanced. Key electronic parameters such as bandgap size, intrinsic doping level, and effective mass of carriers are extracted, providing information for future research based on this system. Through theoretical analysis, we attribute this versatile tunability of the system to the unique crystal structure where the conductive Bi‐O layers and dopant Se/Cl layers are spatially separated. This enables independent optimization of interlayer coupling to tune the bandgap in a wide range, from 0.8 to 3.5 eV, while preserving the intralayer electronic band features. Our result not only opens up a new avenue for further exploitation of Bi_2_O_2_Se‐based materials, but also sets up an example of how targeted atom substitution can effectively modify the electronic structure of a quantum system.

## 2 Result

Illustrated in Figure 1a, the crystal structure of Bi_2_O_2_Se features adjacent conductive Bi‐O layers bridged by Se atoms through ionic bonding. Chlorine substitution turns a single Se layer into two Cl layers and establishes van der Waals connections instead. This modification results in Bi_6_O_6_Se_2_Cl_2_ adopting a 1:2 superlattice structure in the P4/nmm symmetry (Bi_2_O_2_Se‐Bi_2_O_2_Cl_2_‐Bi_2_O_2_Se) with lattice parameters of a = b = 3.89 Å, c = 19.6 Å. The introduction of the van der Waals gap enables facile mechanical exfoliation with standard Scotch tape. As demonstrated in Figure 1b, the optical image and atomic force microscopy (AFM) scanning reveal the exfoliated thin flakes with different thicknesses, where the step edges corresponding to 1‐ and 2‐unit cells (2 and 4 nm, respectively) are evidenced. This demonstrates the feasibility of layer engineering and heterostructure fabrication based on this system. First‐principles calculations predict the preservation of key electronic features with enhanced two‐dimensionality (Figure 1d). The system maintains the indirect bandgap characteristic of Bi_2_O_2_Se, with the conduction band minimum located at Γ and valence band maximum at X. Significantly, the near‐identical band dispersions along both the Γ (blue) and Z (red) planes, corresponding to different *k_z_* values in the Brillouin Zone (Figure 1c), demonstrate markedly reduced out‐of‐plane dependence compared to pristine Bi_2_O_2_Se [2]. This suggests a quasi‐2D electronic behavior in the modified structure.

**FIGURE 2:**
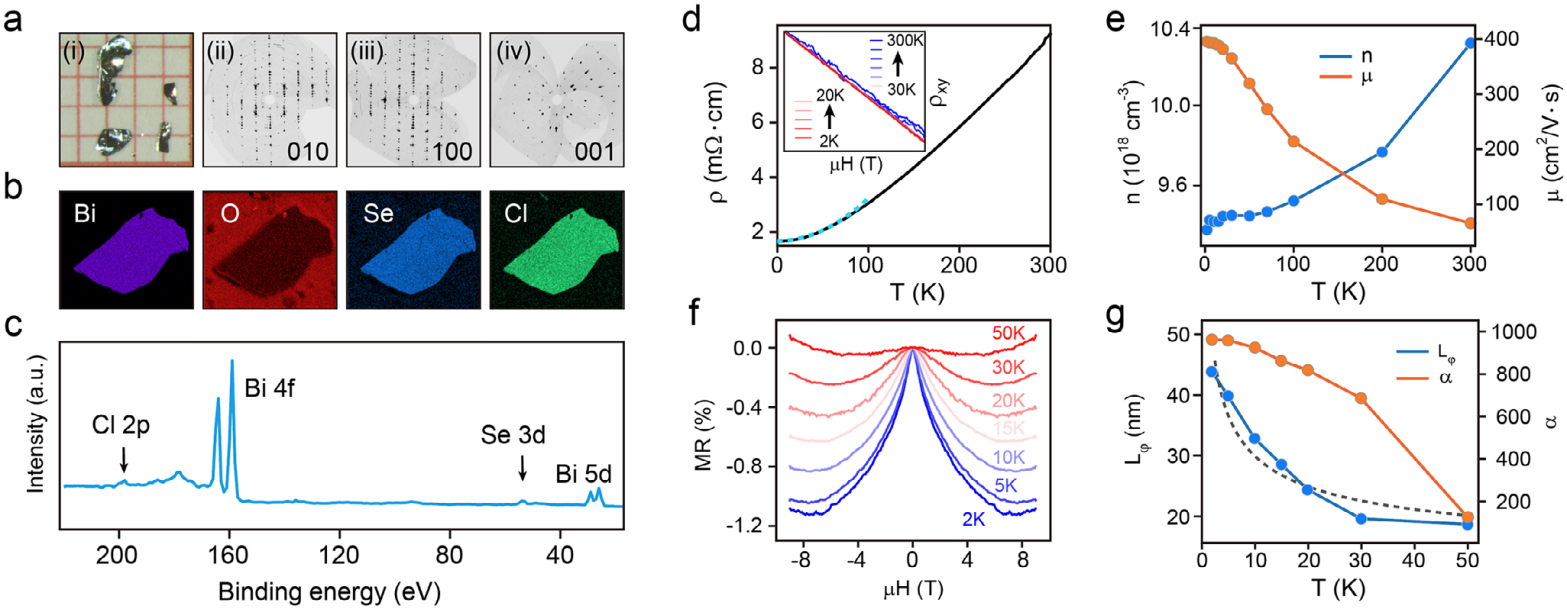
Crystal quality and transport measurements of Bi_6_O_6_Se_2_Cl_2_. (a) (i) Image of high‐quality Bi_6_O_6_Se_2_Cl_2_ single crystal. (ii‐iv) XRD patterns along (010), (100), and (001) surfaces. (b) EDX image of exfoliated Bi_6_O_6_Se_2_Cl_2_ flakes showing spatial uniformity of the elements. (c) Core‐level photoemission spectrum, showing the characteristic peaks of Bi, Se, and Cl core levels. a.u., arbitrary units. (d) Longitudinal resistivity ρ_*xx*_ as a function of temperature of Bi_6_O_6_Se_2_Cl_2_ single crystal. The dotted line is the fitting at low temperature using ρ_*xx*_ = ρ_0_  + *AT* ^2^. Inset: Hall resistivity ρ_*xy*_ as functions of out‐of‐plane magnetic field with temperatures from 2 to 300 K. (e) Extracted carrier densities *n* and carrier mobility μ as a function of temperature. (f) Magnetoresistance (MR) of out‐of‐plane magnetic field with varying temperatures. (g) Extracted phase coherence length *L*_φ_ and prefactor α as a function of temperature under a field limit of 3T. The dotted line is the power‐law fitting of the coherence length, which yields $L_{\varphi }∼T^{-0.42}$.

High‐quality Bi_6_O_6_Se_2_Cl_2_ single crystals (Figure 2a) used for this study were synthesized via the chemical vapor transport (CVT) method (see Methods for details), where the crystal structure is verified through X‐ray diffraction (XRD) measurement. Energy‐dispersive x‐ray spectroscopy (Figure 2b and Figure S1) reveals stoichiometric control and spatial homogeneity, showing a uniform Bi:Cl:Se ratio of 3:1:1 (±1%) across the samples, while x‐ray photoelectron spectroscopy (Figure 2c and Figure S2) identifies the characteristic binding energies of each element, confirming their chemical states.

An overview of the general electronic behavior of this system can be obtained through electronic transport measurements. As shown in Figure 2d, the longitudinal resistivity ρ_*xx*_ of Bi_6_O_6_Se_2_Cl_2_ crystals exhibits metallic behavior, similar to that of the parent compound Bi_2_O_2_Se. This arises from the self‐modulation doping effect of Se/Cl vacancies that populate the conduction band with additional electrons [22, 23]. Consequently, low‐temperature transport reflects heavy carrier doping rather than intrinsic thermal activation across the bandgap. Temperature‐dependent resistivity ρ_*xx*_ can be well fitted by *T* ^2^ (ρ_*xx*_ = ρ_0_  + a*T ^2^, where *ρ*_0_ is the resistivity at 0 K and *a* is a constant) from 2 to 60 K (see Figure 2d), which is a feature of Landau‐Fermi liquid [24, 25], suggesting a sole electron‐electron scattering mechanism in the electrical transport at low temperature (<60 K). Hall measurement results (inset of Figure 2d) corroborate this n‐type conduction, showing the characteristic negative slope that yields an electron density of n = 9.39 × 10^18^ cm^−3^ and a carrier mobility of µ = 395.46 cm^2^ V^−1^ s^−1^ at 2 K (Figure 2e). While this mobility is reduced compared to pristine Bi_2_O_2_Se—likely due to enhanced electron scattering from Cl/Se antisite defects [18] in the current crystals (evidence discussed in Note S2 and Tables S1–S4)—it still surpasses most conventional 2D semiconductors [26] like MoS_2_, highlighting the material's potential for device applications. At low temperatures, the magnetoresistance (MR) shows a characteristic weak localization (WL) cusp below 50 K (Figure 2f), a signature of coherent electron interference effects that may arise from the existence of a strong spin‐orbit interaction [27]. Quantitative fitting analysis (Figure S3 and the result in Figure 2g) of the magnetoconductance using the Hikami‐Larkin‐Nagaoka model (HLN) [28] reveals several intriguing aspects: the extracted phase coherence length (*L*_φ_) of 43.86 nm at 2 K demonstrates well‐preserved quantum effects, while the temperature‐dependent suppression of this length follows expected trends for disordered systems [29, 30]. Notably, *L*_φ_ decreases with increasing temperature and follows a power law of $L_{\varphi }∼T^{-0.42}$. This behavior, together with the prefactor value of α ∼10^2^ that falls between theoretical predictions for pure 2D and 3D systems [31, 32, 33], suggests our engineered superlattice structure achieves a quasi‐2D electronic environment with enhanced quantum confinement.

**FIGURE 3:**
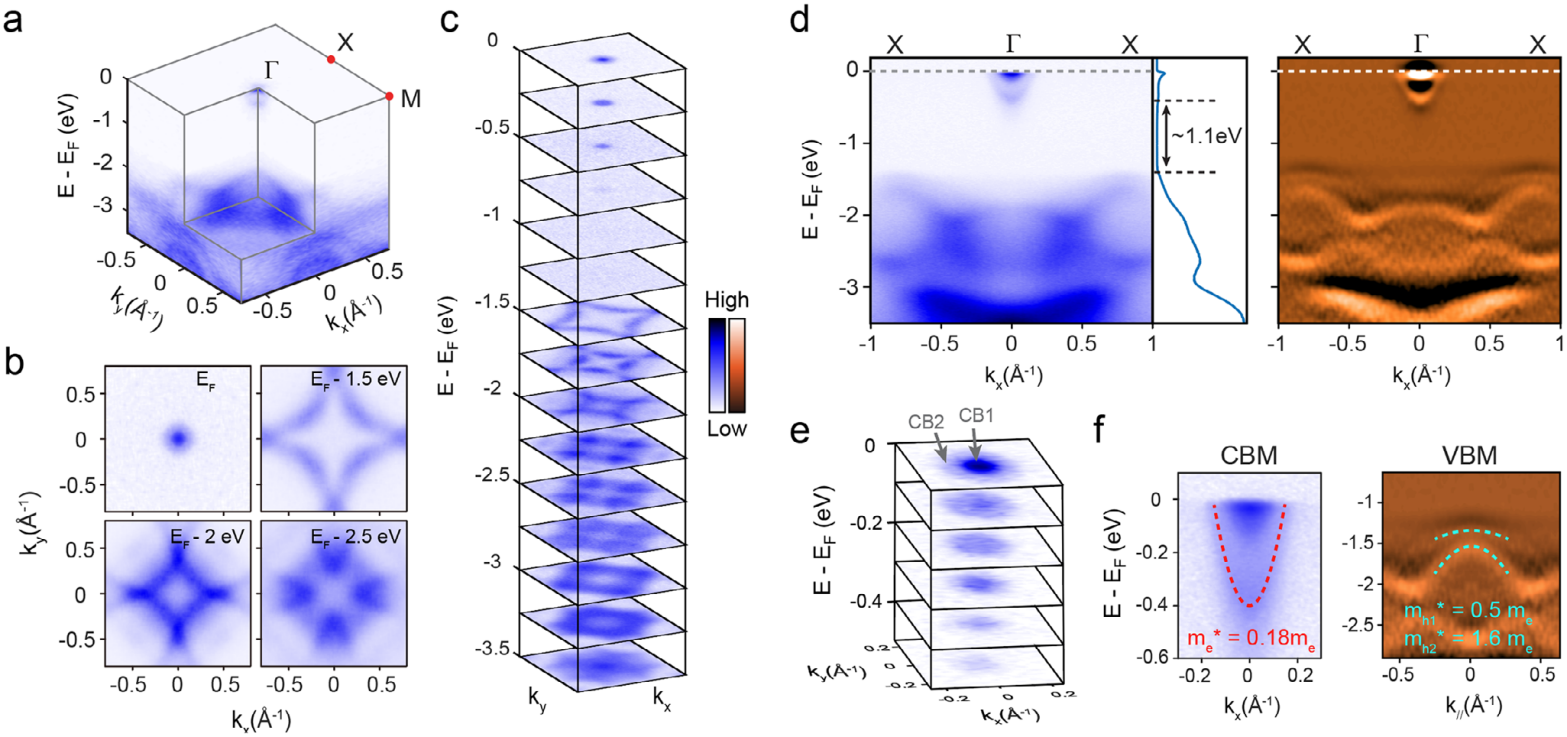
General electronic band structure of Bi_6_O_6_Se_2_Cl_2_. (a) 3D plot of the photoemission intensity measured on Bi_6_O_6_Se_2_Cl_2_ single crystal. Conduction band bottom and valence band top is evidenced at Γ and X point respectively. (b) Constant energy contours of the photoemission intensity on Fermi level (*E_F_*) and at binding energies of −1.5, −2, and −2.5 eV, respectively. (c) Stacking plots of constant energy contours showing the complete band structures. (d) Band dispersion cut and its second derivative plot showing the band structure along X − Γ − X direction. The extracted density of state (DOS) is plotted on the side, illustrating an indirect bandgap of ∼1.1 eV. (e) Stacking plot zoomed in to the two conduction bands (CB), with detailed dispersion shown in f. (f) Fitting of the conduction band minimum (CBM) and valence band maximum (VBM) along Γ − X direction.

To elucidate the detailed electronic band structure of the Bi_6_O_6_Se_2_Cl_2_ system, synchrotron‐based high‐resolution ARPES measurements were performed on the 001‐cleavage plane of the crystals. From the 3D overview of the ARPES intensity (Figure 3a), the conduction band minimum (CBM) is evidenced at the Γ point, while the valence band maximum (VBM) was found at the X point, confirming the indirect bandgap nature of the system. The observed four‐fold symmetry of the valence bands, as evident in the constant energy contours (Figure 3b,c), is in line with the lattice symmetry. These general electronic features match closely with the parent compound Bi_2_O_2_Se, suggesting that chlorine substitution preserves the basic electronic structure. A detailed examination along the high‐symmetry direction Γ‐X‐Γ (Figure 3d) reveals an indirect bandgap size of 1.10 ± 0.07 eV. Notably, the valence band edge exhibits slight broadening, which likely stems from the disorder effects induced by Se/Cl antisite defects [17, 34, 35], as these states are primarily derived from Se/Cl p‐orbitals [17]. In contrast, the conduction band remains sharp. Zoomed‐in analysis (Figure 3e) reveals a self‐doping level of ∼ 0.4 eV, with two isotropic electron pockets observed. The effective mass of the electrons, obtained via parabolic fitting to the band dispersion (Figure 3f), yields m_e_* ∼ 0.18 ± 0.03 m_0_ (m_0_ is the mass of a free electron). In addition, two distinct hole pockets, one heavy and one light, coexist at the VBM. Their effective masses along the Γ‐X direction are estimated to be m_h1_* ∼ −0.50 ± 0.12 m_0_, and m_h2_* ∼ −1.60 ± 0.06 m_0_, respectively. This coexistence of light and heavy hole bands makes the system a promising candidate for thermoelectric applications, as the light holes facilitate high electrical conductivity while the heavy holes help maintain a large Seebeck coefficient [21, 36].

**FIGURE 4:**
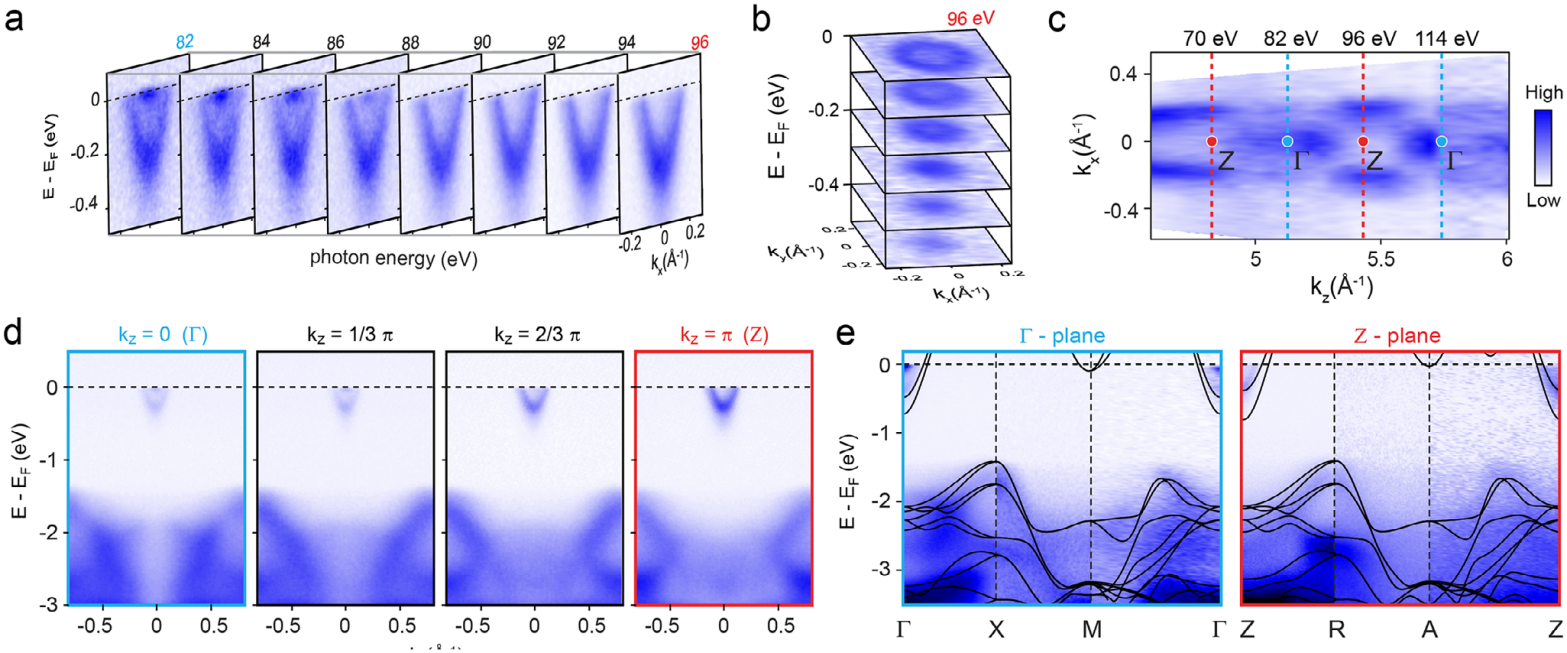
Detailed quasi‐2D electronic features in full Brillouin Zone. (a) Photoemission intensity of electron pockets with varying photon energies from 82 eV (Γ‐plane) to 96 eV (Z‐plane), showing its evolution with out‐of‐plane momentum (*k_z_*). (b) Stacking plot of the electron pocket on the Z‐plane. (c) Fermi surface of *k_x_‐k_z_* plane, showing the periodic evolution of two conduction bands. High symmetry positions Γ and Z are labeled. (d) High statistic cuts of the band dispersion along X − Γ − X direction at different *k_z_* value of 0, π/3, 2π/3, π respectively, showing almost identical features. (e) Detailed band dispersion along high symmetry directions on both the Γ‐plane and Z‐plane with DFT calculation overlaid, indicative of the quasi‐2D nature of the system.

To evaluate the quasi‐2D nature of the system, we further performed photon‐energy‐dependent ARPES measurements to probe the electronic structure across different *k_z_* values within the 3D Brillouin zone (BZ). Theoretical calculations predict a *k_z_*‐dependent evolution of the electron pocket (Figure 1d), characterized by a varying energy separation between the two conduction bands between the Γ and Z planes. As illustrated in Figure 4a, tuning the photon energy from 82 to 96 eV reveals that while the lower conduction band remains nearly stationary, the upper conduction band vanishes, as can be more clearly visualized in the constant energy contours of Figure 4b. A wide‐range photon energy scan covering multiple BZs (Figure 4c) confirms the periodic reappearance of this upper conduction band, allowing for the precise identification of the high‐symmetry Γ (blue) and Z (red) planes along the *k_z_* direction (see Note S1 for detailed analysis). Despite the *k_z_*‐sensitivity of the upper conduction band, the primary electronic features exhibit remarkable similarity between the Γ and Z planes. This is demonstrated by the band dispersion cuts at *k_z_* = 0, π/3, 2π/3, and π (Figure 4d), which contrast sharply with the more dispersive nature of pristine Bi_2_O_2_Se^2^. A direct comparison of the dispersions and overlaid DFT calculations at the Γ and Z points (Figure 4e) shows nearly identical features. These results signify successful electronic structure engineering, effectively enhancing the two‐dimensionality of the system while preserving its fundamental electronic properties.

## 3 Discussion

**FIGURE 5:**
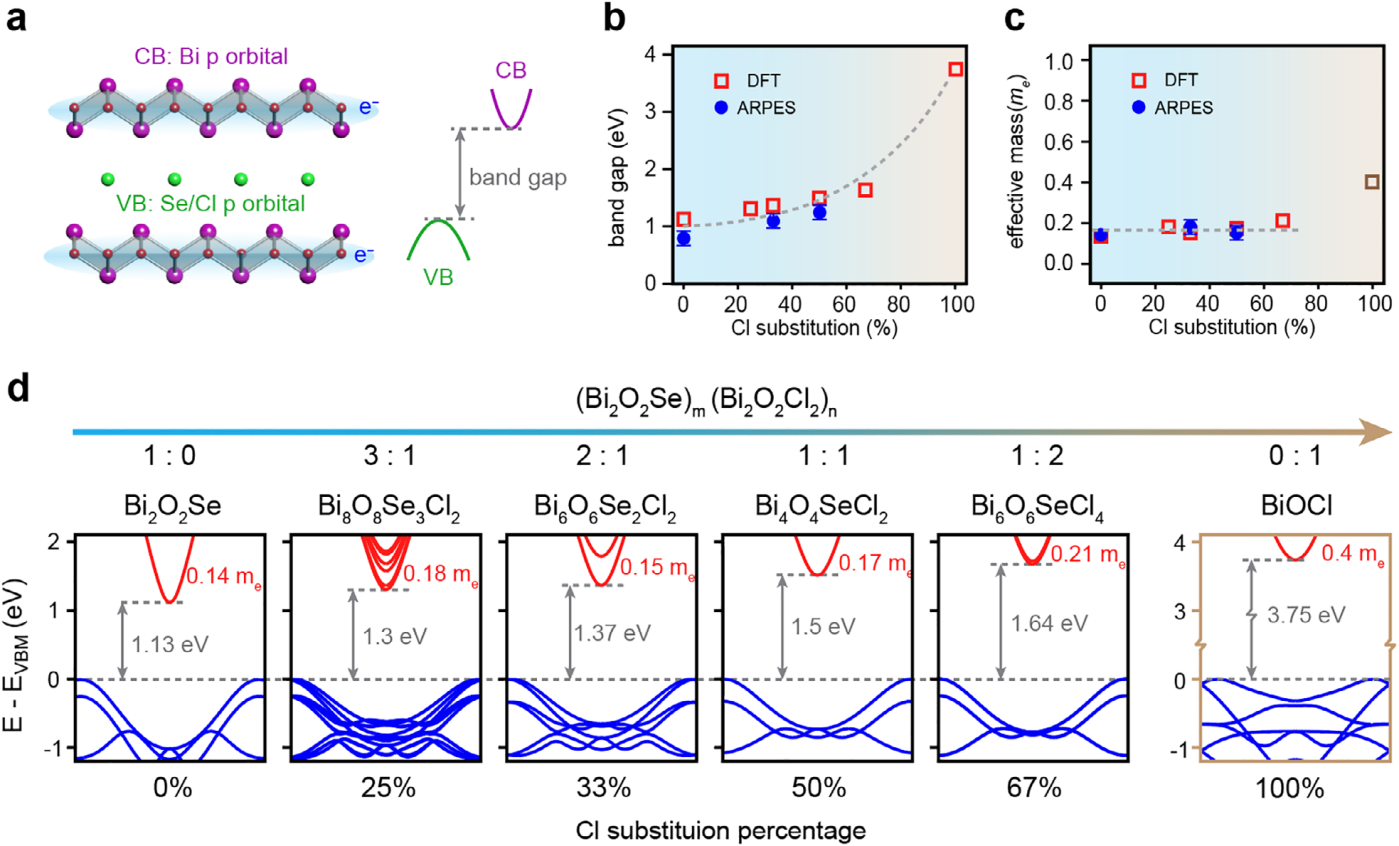
Rational band structure engineering with Cl substitution in Bi_2_O_2_Se family. (a) Schematic showing the spatial separation of the conductive Bi‐O layer and the dopant Se/Cl layer. The conduction band mainly consists of Bi p orbitals, while the valence bands are contributed by Se/Cl p orbitals. (b,c) Evolution of the indirect bandgap size and effective mass of the conduction band (derived from both DFT calculation and ARPES experiments, summarized data in Table 1) as a function of Cl substitution over Se. With the weakening of the interlayer interaction in Se/Cl layer, the bandgap size of the system increases monotonically, while the pristine conduction band characteristics from Bi‐O layer (e.g. effective mass of the electron pocket) are preserved. Data for Bi_2_O_2_Se is from Ref [2]. (d) Detailed band dispersion along X − Γ − X direction as a function of Cl substitution, showing systematic evolution.

The exceptional tunability of Bi_2_O_2_Se through chlorine substitution originates from its unique layered structure and orbital characteristics. As illustrated in Figure 5a, the system exhibits a natural electronic decoupling where conduction electrons, primarily derived from Bi p orbitals, remain strongly localized within the conductive Bi‐O layers, while Se vacancies in the adjacent layers provide self‐doping electrons that make the system intrinsically metallic [22]. This spatial separation creates a remarkable protection of the electrons from the disorder scatterings in the Se/Cl layers, resulting in a high carrier mobility of the system. The introduction of van der Waals gaps through Cl substitution maintains this scenario and further produces two complementary effects. It systematically reduces interlayer hybridization, enhancing two‐dimensionality and thereby driving a monotonic widening of the bandgap (∼0.8 to ∼ 3.5 eV), as indicated by the result of DFT calculations and experimental verification (Figure 5b,c and Table 1). On the other side, it preserves the pristine conduction band characteristics, such as band dispersion and effective mass (Figure 5b,c). These two effects are confirmed with consistent experimental measurements and theoretical calculations (Figure 5b), illustrating how spatial separation of functional sublattices allows for independent tuning of interlayer coupling while preserving desirable intralayer characteristics in this system. Detailed calculation showing the band structure evolution is presented in Figure 5d, and the ARPES measurement on high‐quality single crystal Bi_4_O_4_SeCl_2_ (50% substitution) is presented in Figures S4 and S5.

**TABLE 1:** Extracted band parameters from ARPES spectra. m_e_^*^/m_h_^*^: Effective mass of electron/hole pockets in the unit of free electron m_0_. Data of Bi_2_O_2_Se is adapted from Ref 2.

	m_e_^*^	m_h_^*^	bandgap
**Bi_2_O_2_Se (Cl 0%)**	(0.14 ± 0.02) m_0_	(‐2.41 ± 0.02) m_0_	(0.80 ± 0.05) eV
**Bi_6_O_6_Se_2_Cl_2_ (Cl 33%)**	(0.18 ± 0.03) m_0_	(‐0.50 ± 0.12) m_0_ (h1) (‐1.60 ± 0.06) m_0_ (h2)	(1.10 ± 0.07) eV
**Bi_4_O_4_SeCl_2_ (Cl 50%)**	(0.16 ± 0.02) m_0_	(‐1.2 ± 0.1) m_0_	(1.25 ± 0.07) eV

The unique electronic properties of chlorine‐substituted Bi_2_O_2_Se offer significant potential for diverse industrial applications. Specifically, the widely tunable bandgap and van der Waals (vdW) interactions enable the fabrication of photodetectors with high responsivity, high stability, continuous broadband tuning, simple manufacturing, and low cost [9, 10, 11]. The preserved high electron mobility facilitates high‐performance, fast‐response, nanoelectronic devices with low power consumption. Moreover, the coexistence of light and heavy hole bands, with suppressed phonon‐mediated heat transport, positions this material family as a promising candidate for next‐generation thermoelectric devices [21, 36]. Meanwhile, multiple approaches should be applied to further optimize the quality of these Cl‐substituted crystals. The current mobility limitations, evident from valence band smearing (Figure 3d), primarily originate from Se‐Cl antisite defects, which could be suppressed with optimization of growth parameters, specifically the tuning of Se/Cl partial pressures and the implementation of slower post‐growth annealing cycles. Such enhancements would simultaneously benefit practical device performance and enable more precise investigations of quantum transport phenomena. Notably, this band structure engineering approach has broader implications beyond halogen substitution. Following the paradigm established in TaS_2_ and related dichalcogenides family, where intercalation has unveiled rich phenomena including magnetic ordering, topological states, and unconventional superconductivity etc [37, 38, 39], we anticipate Bi_2_O_2_Se to be a versatile platform for discovering novel quantum phenomena through similar structural modifications.

## 4 Methods

### 4.1 Sample Synthesis and Basic Characterizations

Bi_6_O_6_Se_2_Cl_2_ single crystals were grown by CVT with iodine (I_2,_ Alfa‐Aesar, 99.9985%) as the transport agent. We first synthesized Bi_6_O_6_Se_2_Cl_2_ polycrystalline powders by the direct stoichiometric solid‐state reaction of high pure Bi_2_O_3_ powder (Sinopharm Chemical Reagent Co., Ltd, 99.999%), BiOCl powder (Alfa Aesar, 99.99%), bismuth powder (Alfa Aesar, 99.999%) and selenium powder (Alfa Aesar, 99.999%) with a 2:6:2:3 molar ratio in a fused silica tube sealed under vacuum (P ∼ 4 × 10^−6^ Torr) at about 850°C for several days. Then the mixture of Bi_6_O_6_Se_2_Cl_2_ and I_2_ (about 10 mg/mL) powders were loaded into the sealed evacuated quartz tube (P ∼ 4 × 10^−6^ Torr), and then put into a two‐zone furnace to grow crystals. The raw materials were placed at the hot zone of the ampoule to maintain crystal growth and the empty end of the quartz tube was maintained at a lower temperature to grow crystals. The millimeter‐sized crystals with metallic luster were obtained successfully after growing at a temperature profile of 750°C–650°C for over 14 days. To determine the surface orientation of the Bi_6_O_6_Se_2_Cl_2_ single crystals, x‐ray diffraction (XRD) measurements were conducted on an x‐ray diffractometer (Ultima III Rigaku) using Cu‐*K*_α_ radiation with a 2θ range of 5∼85°. The composition of single crystals was characterized by an energy‐dispersive spectroscopy (EDS) spectrometer equipped with a scanning electron microscope (FEI Inc., Quanta). Longitudinal resistivity *ρ_xx_* and Hall (transversal) resistivity *ρ_xy_* were measured using a standard four‐probe configuration on a 9 T Quantum Design Physical Property Measurement System (PPMS).

### 4.2 Angle‐Resolved Photoemission Spectroscopy (ARPES)

ARPES measurements were performed at BL03U of the Shanghai Synchrotron Radiation Facility (SSRF) [40]. The data were recorded using a DA30L analyzer with the total convolved energy and angle resolutions of 10 meV and 0.2°, respectively. The samples were cleaved in situ on the (001) cleavage surface and the measurements were carried out under ultrahigh vacuum of better than 1.3 × 10^−10^ mbar at the temperature of 12 K.

### 4.3 Theoretical Calculation

The band structure was calculated by density functional theory in the generalized gradient approximation implemented in the Vienna ab‐initio simulation package (VASP) [41]. The projected augmented wave method and the Perdew‐Burke‐Ernzerhof exchange correlation are used in the calculations within the generalized gradient approximation (GGA) [42]. The plane‐wave cutoff energy was set to 520 eV, with the Brillouin zone sampled by a 9 × 9 × 9, 6 × 6 × 6, 8 × 8 × 2, 10 × 10 × 1, and 9 × 9 × 6 k‐grid for Bi_2_O_2_Se, Bi_4_O_4_SeCl_2_, Bi_6_O_6_Se_2_Cl_2_, Bi_6_O_6_SeCl_4_/Bi_8_O_8_Se_3_Cl_2_, and BiOCl, respectively. Except for Bi_2_O_2_Se, the optB86b‐vdW correlation functional has been used in the calculations. To obtain accurate bandgaps, we also employ the Heyd‐Scuseria‐Ernzerhof (HSE06) hybrid functional in the band structure calculations [43, 44].

## Author Contributions

**Yong‐Zhen Jiang**: data curation, Writing – original draft, investigation, validation, formal analysis, visualization. **Qi‐Xun Wen**: data curation, validation, investigation, formal analysis, visualization. **Xiang Xu**: validation, formal analysis. **Su‐Tao Sun**: data curation, investigation, visualization. **Feng‐Fan Ren**: data curation. **Zi‐Xuan Zhou**: data curation. **Xing‐Yuan You**: data curation, investigation. **Yu‐Cheng Kan**: data curation, investigation. **Zheng Zhang**: investigation, formal analysis. **Jie‐Yi Liu**: validation, investigation. **Yu‐Zhou Zhao**: validation, investigation. **Zheng‐Tai Liu**: data curation, methodology. **Mao Ye**: methodology, data curation. **Shu‐Hua Yao**: supervision, investigation. **Jian Zhou**: supervision, investigation, formal analysis, validation. **Yan‐Bin Chen**: supervision, validation. **Zhong‐Kai Liu**: supervision, validation. **Yu‐Lin Chen**: validation, supervision. **Yang‐Yang Lv**: conceptualization, validation, formal analysis, supervision, resources, writing – review and editing, writing – original draft, funding acquisition, visualization, investigation. **Cheng Chen**: conceptualization, validation, formal analysis, supervision, resources, writing – original draft, writing – review and editing, funding acquisition, visualization, investigation.

## Conflicts of Interest

The authors declare no conflicts of interest.

## Supporting information




**Supporting File**: advs77919‐sup‐0001‐SuppMat.docx.

## Data Availability

The data that support the findings of this study are available from the corresponding author upon reasonable request.
