# Supplementary material for: Enhancing Quasi‐Two‐Dimensional Electronic Structure in Bi2O2Se via Chlorine Substitution
Source: Adv Sci (Weinh). 2026 Sep 27:e77919. Online ahead of print. doi: 10.1002/advs.77919 (PMC13616381; doi:10.1002/advs.77919)
Supplement: Supplementary file 1 [file ADVS-9999-e77919-s001.docx]

**Supplementary Information for**

**“****Enhancing Quasi-Two-Dimensional Electronic Structure in Bi_2_O_2_Se via Chlorine Substitution****”**

Yong-Zhen Jiang^1*^, Qi-Xun Wen^2*^, Xiang Xu^1*^, Su-Tao Sun^2*^, Feng-Fan Ren^1^, Zi-Xuan Zhou^1^, Xing-Yuan You^2^, Yu-Cheng Kan^2^, Zheng Zhang^1^, Jie-Yi Liu^3^, Yu-Zhou Zhao^1^, Zheng-Tai Liu^4,5^, Mao Ye^4,5^, Shu-Hua Yao^2^, Jian Zhou^2^, Yan-Bin Chen^2^, Zhong-Kai Liu^1^, Yu-Lin Chen^1^, Yang-Yang Lv^2†^, Cheng Chen^1†^

*^1^State Key Laboratory of Quantum Functional Materials and ShanghaiTech Laboratory for Topological Physics, School of Physical Science and Technology, ShanghaiTech University, Shanghai,201210, China*

*^2^ National Laboratory of Solid State Microstructures, Department of Materials Science and Engineering, Department of Physics, Nanjing University, Nanjing, 210093, China*

*^3^Diamond Light Source, Harwell Science and Innovation Campus, Didcot OX11 0DE, UK*

*^4^Shanghai Synchrotron Radiation Facility, Shanghai Advanced Research Institute, Chinese Academy of Sciences, Shanghai, 201210, China*

*^5^National Key Laboratory of Materials for Integrated Circuits, Shanghai Institute of Microsystem and Information Technology, Chinese Academy of Sciences, Shanghai 200050, China*

**These authors contributed equally to the work*

*† Correspondence to:* [*lvyangws0801@nju.edu.cn*](mailto:lvyangws0801@nju.edu.cn)*,* [*chencheng1@shanghaitech.edu.cn*](mailto:chencheng1@shanghaitech.edu.cn)

**Table of contents:**

**Note 1 | Determination of k_z_ value in ARPES experiment**

**Fig. S1 | EDX intensity of exfoliated Bi_6_O_6_Se_2_Cl_2_ flakes**

**Fig. S2 | Peak fitting of the XPS spectra of Bi_6_O_6_Se_2_Cl_2_ single crystals**

**Fig. S3 |** **Magnetoconductance and fitting**

**Fig. S4 | XRD and Laue data for high-quality Bi_4_O_4_SeCl_2_ single crystal**

**Fig. S5 | ARPES measurement on Bi_4_O_4_SeCl_2_ single crystal**

**Note 2 | Determination of antisite defect from XRD measurement**

**Table S1-2 | XRD refinement results for Bi_4_O_4_SeCl_2_**

**Table S3-4 | XRD refinement results for Bi_6_O_6_Se_2_Cl_2_**

**Note 1 | Determination of k_z_ value in ARPES experiment**

During the photoemission process, the horizontal momentum (k**_//_**) of band electrons is obtained directly from the free electrons based on the momentum conservation law. The vertical momentum (k_z_) is not conserved. With the free-electron final state approximation and a potential parameter V_0_ (also known as the inner potential) describing the energy difference of photoelectrons before and after passing the crystal surface, we can derive the k_z_ as

$$k_{z}= \frac{\sqrt{2m_{e}(E_{k} {cos}^{2}\theta+ V_{0})}}{\hbar}$$

where θ is the emission angle and E_k_ is the kinetic energy of the emitted electron, which satisfies E_k_ = $h\nu$ − $w$ − E_B_ where $h\nu$ is the photon energy, $w$ is the work function of the sample, and EB is the electron binding energy. As V_0_ varies with compounds, we typically perform energy-dependent ARPES by using a broad range of photon energies to ensure the full coverage of k_z_–span (ideally more than one Brillouin Zone (BZ)), and then use the high-symmetry points in the k||-k_z_ plane of the BZ to identify the exact value of V_0_. The inner potential for the measurement on Bi_6_O_6_Se_2_Cl_2_ (Fig. 4 in the main text) was determined to be V_0_ = 17 eV.

**Fig. S1|** **EDX intensity of exfoliated Bi_6_O_6_Se_2_Cl_2_ flakes.** Integrated intensity of EDX spectrum, where the ratio of the elements is extracted.

**Fig. S2|** **Peak fitting of the XPS spectra of Bi_6_O_6_Se_2_Cl_2_ single crystals**. Spectra of Bi 4*f*, Se 3*d*, and O 1*s* orbitals, confirming the chemical states of the elements.

**Fig. S3|** **Magnetoconductance and fitting.** Low-field (from -3 T to 3 T) magnetoconductance $\Delta G=G\left( H \right)-G(0)$ curves at a series of temperatures. The Hikami-Larkin-Nagaoka (HNL) model fitting (black lines) takes the form of $\Delta G\left( H \right)=\frac{\alpha e^{2}}{2\pi^{2}\hbar}\left[ \psi\left( \frac{1}{2}+\frac{\hbar}{4eHL_{\psi}} \right)-\ln\left( \frac{\hbar}{4eHL_{\psi}} \right) \right]$, where *e* represents the electron charge and *ћ* denotes the reduced Planck constant. The digamma function $\psi$ is involved, along with the phase coherence length $L_{\psi}$, and *α* is the prefactor.

**Fig. S4|** **XRD and Laue data for high-quality Bi_4_O_4_SeCl_2_ single crystal.**

**Fig. S5|** **ARPES measurement on Bi_4_O_4_SeCl_2_ single crystal. a.** The 3D Brillouin Zone (BZ) of Bi_4_O_4_SeCl_2,_ and the blue frames (overlapped) show the k_y_-k_z_ plane. **b,** Constant energy contour at E_F_ in the k_||_ and k_z_ planes of Bi_4_O_4_SeCl_2_, in which high symmetry points are labeled. (c) General band evolution among high symmetry points, showing excellent agreement with theoretical calculation. (d) The directions of ARPES mapping are along $\bar{X}-\bar{\Gamma}-\bar{X}$ . (e) Photon emission spectrum of the electron pocket located around the Z point, as well as the fitting result. The yellow solid line indicates the parabolic fitting result.

**Note 2 | Determination of antisite defect from XRD result**

Single-crystal X-ray diffraction data were collected on a Bruker APEX-II CCD diffractometer using Mo Kα radiation λ = 0.71073 Å at 100 K. A suitable single crystal was selected and mounted on the diffractometer, and data were collected using combined φ and ω scan modes. Data reduction, including Lorentz and polarization corrections, was carried out using SAINT, and multi-scan absorption correction was performed with SADABS. The structure was solved using SHELXT and refined by full-matrix least-squares refinement on F² using SHELXL within the Olex2 graphical interface. All non-hydrogen atoms were refined anisotropically.

The results show that Bi_4_O_4_SeCl_2_ and Bi_6_O_6_Se_2_Cl_2_ both belong to the tetragonal crystal systems, with the space groups of I4/mmm (No. 139) and P4/nmm (No. 129), respectively. Their chemical formulas are determined as Bi_4_O_4_Se_1.06_Cl_1.94_ and Bi_6_O_6_Se_2_Cl_2_. Notably, the Se and Cl atoms exhibit occupational disorder over two crystallographic anion sites in Bi_4_O_4_Se_1.04_Cl_1.96_. The refinement results of Bi_4_O_4_Se_1.04_Cl_1.96_ in Table S2 show that the 2a site is occupied by Se/Cl with a ratio of 0.54/0.46, whereas the 4e site is occupied by Se/Cl with a ratio of 0.26/0.74. This mixed occupancy corresponds to coupled Se/Cl antisite disorder, namely Cl_Se_ and Se_Cl_ defects. A similar occupational disorder is observed in Bi_6_O_6_Se_2_Cl_2_. The refinement results in Table S4 show that the Se and Cl atoms are occupationally disordered over two 2c anion sites. The first site is predominantly occupied by Se with a Se/Cl ratio of 0.75/0.25, whereas the second site is predominantly occupied by Cl with a Se/Cl ratio of 0.25/0.75. Overall, our result shows that Se/Cl antisite defects exist in both two materials.

**Table S1. Single-crystal X-ray refinement results for Bi_4_O_4_Se_1.04_Cl_1.96_ and structure refinement for Bi_4_O_4_Se_1.04_Cl_1.96_**

| Identification code | Bi_4_O_4_SeCl_2_ |
| --- | --- |
| Empirical formula | Bi_4_O_4_Se_1.04_Cl_1.96_ |
| Temperature, K | 100 |
| Formula weight | 1052.61 |
| Wavelength, Å | 0.71073 |
| Crystal system, space group | tetragonal, *I*4/mmm(No. 139) |
| *a*, Å | 3.8989(6) |
| *b*, Å | 3.8989(6) |
| *c*, Å | 27.039(5) |
| *a*=*g=b*, deg | 90 |
| Volume, Å^3^ | 415.42(15) |
| *Z* | 2 |
| Calculated density, g/cm^−3^ | 8.50444 |
| Absorption coefficient, mm^−1^ | 90.672 |
| *F*(000) | 866.0 |
| Crystal size, mm^3^ | 0.12×0.06×0.02 |
| Completeness to theta = 25.242 | 96.1% |
| GOF on *F*^2^ | 1.140 |
| *R*1, *wR*2 [I >2*σ*(I)] | *R*1 = 0.0287, *wR*2 = 0.0704 |
| *R*1, *wR*2 (all data) | *R*1 = 0.0295, *wR*2 = 0.0707 |
| *R*_1_ *=* ∑\|\|*F_o_*\|-\|*F_c_\|*\|/∑\|*F_o_*\|, *wR*_2_ ={∑*w*[(*F_o_*)^2^ -(*F_c_*)^2^]^2^/∑*w*[(*F_o_*)^2^]^2^}*^1/2^* | |

**Table S2. Atomic coordinates (**$\boldsymbol{\times}$**10^4^) and equivalent isotropic displacement parameters (Å^2^** $\boldsymbol{\times}$**10^4^) for Bi_4_O_4_Se_1.04_Cl_1.96_.**

**U(eq) is defined as one-third of the trace of the orthogonalized U_ij_ tensor.**

| **Atom** | **x/a** | **y/b** | **z/c** | **U(eq)** | **S.O.F.** | **Wyckoff** |
| --- | --- | --- | --- | --- | --- | --- |
| Bi1 | 1 | 1 | 0.56837 | 12 | 1 | 4e |
| Bi2 | 1/2 | 1/2 | 0.66121(4) | 18.2(3) | 1 | 4e |
| O1 | 1/2 | 1 | 0.611880 | 9 | 1 | 8g |
| Se1 | 1/2 | 1/2 | 1/2 | 12(2) | 0.54(4) | 2a |
| Cl1 | 1/2 | 1/2 | 1/2 | 12(2) | 0.46(4) | 2a |
| Se2 | 0 | 0 | 0.70947(17) | 13(2) | 0.26(3) | 4e |
| Cl2 | 0 | 0 | 0.70947(17) | 13(2) | 0.74(3) | 4e |

**Table S3. Single-crystal X-ray refinement results for Bi_6_O_6_Se_2_Cl_2_** **and structure refinement for Bi_6_O_6_Se_2_Cl_2_**

| Identification code | Bi_6_O_6_Se_2_Cl_2_ |
| --- | --- |
| Empirical formula | Bi_6_O_6_Se_2_Cl_2_ |
| Temperature, K | 100 |
| Formula weight | 1578.7 |
| Wavelength, Å | 0.71073 |
| Crystal system, space group | tetragonal, *P*4/nmm(129) |
| *a*, Å | 3.8946(2) |
| *b*, Å | 3.8946(2) |
| *c*, Å | 19.6602(13) |
| *a*=*g=b*, deg | 90 |
| Volume, Å^3^ | 298.20(4) |
| *Z* | 1 |
| Calculated density, g/cm^−3^ | 8.79041 |
| Absorption coefficient, mm^−1^ | 95.323 |
| *F*(000) | 648.0 |
| Crystal size, mm^3^ | 0.16×0.08×0.01 |
| Completeness to theta = 25.242 | 96.8% |
| GOF on *F*^2^ | 1.171 |
| *R*1, *wR*2 [I >2*σ*(I)] | *R*1 = 0.0287, *wR*2 = 0.0762 |
| *R*1, *wR*2 (all data) | *R*1 = 0.0297, *wR*2 = 0.0768 |
| *R*_1_ *=* ∑\|\|*F_o_*\|-\|*F_c_\|*\|/∑\|*F_o_*\|, *wR*_2_ ={∑*w*[(*F_o_*)^2^ -(*F_c_*)^2^]^2^/∑*w*[(*F_o_*)^2^]^2^}*^1/2^* | |

**Table S4. Atomic coordinates (**$\boldsymbol{\times}$**10^4^) and equivalent isotropic displacement parameters (Å^2^** $\boldsymbol{\times}$**10^4^) for Bi_6_O_6_Se_2_Cl_2_.**

**U(eq) is defined as one-third of the trace of the orthogonalized U_ij_ tensor.**

| **Atom** | **x/a** | **y/b** | **z/c** | **U(eq)** | **S.O.F.** | **Wyckoff** |
| --- | --- | --- | --- | --- | --- | --- |
| Bi1 | 1/4 | 1/4 | 0.74917(4) | 9.4(4) | 1 | 2c |
| Bi2 | 1/4 | 1/4 | 0.56409(4) | 13.7(4) | 1 | 2c |
| Bi3 | 3/4 | 3/4 | 0.87740(5) | 17.8(4) | 1 | 2c |
| O1 | 1/4 | 3/4 | 0.8085(4) | 8(2) | 1 | 4f |
| O2 | -1/4 | 1/4 | 1/2 | 10(3) | 1 | 2b |
| Se1 | 3/4 | 3/4 | 0.65125(12) | 9.6(7) | 0.75 | 2c |
| Cl1 | 3/4 | 3/4 | 0.65125(12) | 9.6(7) | 0.25 | 2c |
| Se2 | 5/4 | 5/4 | 0.9440(2) | 10.6(11) | 0.25 | 2c |
| Cl2 | 5/4 | 5/4 | 0.9440(2) | 10.6(11) | 0.75 | 2c |
